# [*N*,*N*-Bis(2-amino­eth­yl)ethane-1,2-diamine](ethane-1,2-diamine)­nickel(II) thio­sulfate trihydrate

**DOI:** 10.1107/S1600536812001651

**Published:** 2012-01-18

**Authors:** Beatrix Seidlhofer, Christian Näther, Wolfgang Bensch

**Affiliations:** a Institut für Anorganische Chemie, Christian-Albrechts-Universität Kiel, Max-Eyth-Strasse 2, 24118 Kiel, Germany

## Abstract

The title compound, [Ni(C_2_H_8_N_2_)(C_6_H_18_N_4_)]S_2_O_3_·3H_2_O, was accidentally synthesized under solvothermal conditions applying [Ni(en)_3_]Cl_2_ (en is ethane-1,2-diamine) as the Ni source. The asymmetric unit consists of one discrete [Ni(tren)(en)]^2+^ complex [tren is *N*,*N*-bis­(2-amino­eth­yl)ethane-1,2-diamine] in which the Ni^2+^ cation is sixfold coord­inated within a slightly distorted octa­hedron, one thio­sulfate anion and three water mol­ecules. In the crystal, the complex cations, anions and water mol­ecules are linked by an intricate hydrogen-bonding network. One C atom of the tren ligand, as well as one O atom of a water mol­ecule, are disordered over two sites and were refined using a split model (occupancy ratios = 0.85:15 and 0.60:0.40, respectively).

## Related literature

For background of this work, see: Lühmann *et al.* (2011 ▶); Seidlhofer *et al.* (2011 ▶). For related thio­sulfate crystal structures, see: Nardelli & Coghi (1958 ▶); Varand *et al.* (1967 ▶); Freire *et al.* (2000 ▶); Díaz de Vivar *et al.* (2007 ▶).
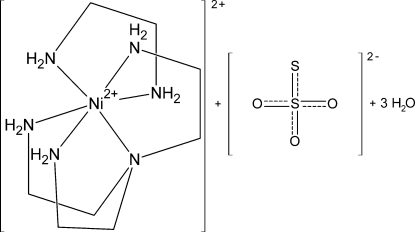


## Experimental

### 

#### Crystal data

[Ni(C_2_H_8_N_2_)(C_6_H_18_N_4_)]S_2_O_3_·3H_2_O
*M* *_r_* = 431.23
Monoclinic, 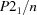

*a* = 10.890 (2) Å
*b* = 10.0494 (17) Å
*c* = 16.689 (3) Å
β = 96.68 (2)°
*V* = 1813.9 (6) Å^3^
*Z* = 4
Mo *K*α radiation
μ = 1.34 mm^−1^
*T* = 170 K
0.16 × 0.11 × 0.06 mm

#### Data collection

Stoe IPDS-1 diffractometer
Absorption correction: numerical (*X-SHAPE*; Stoe & Cie, 1998 ▶) *T*_min_ = 0.559, *T*_max_ = 0.748
23884 measured reflections
4358 independent reflections
3924 reflections with *I* > 2σ(*I*)
*R*_int_ = 0.065

#### Refinement

*R*[*F* ^2^ > 2σ(*F* ^2^)] = 0.037
*wR*(*F* ^2^) = 0.099
*S* = 1.05
4358 reflections
216 parameters
2 restraints
H-atom parameters constrained
Δρ_max_ = 0.55 e Å^−3^
Δρ_min_ = −0.77 e Å^−3^

### 

Data collection: *IPDS Program Package* (Stoe & Cie, 1998 ▶); cell refinement: *IPDS Program Package*; data reduction: *IPDS Program Package*; program(s) used to solve structure: *SHELXS97* (Sheldrick, 2008 ▶); program(s) used to refine structure: *SHELXL97* (Sheldrick, 2008 ▶); molecular graphics: *SHELXTL* (Sheldrick, 2008 ▶) and *DIAMOND* (Brandenburg, 2006 ▶); software used to prepare material for publication: *SHELXTL*.

## Supplementary Material

Crystal structure: contains datablock(s) I, global. DOI: 10.1107/S1600536812001651/wm2582sup1.cif

Structure factors: contains datablock(s) I. DOI: 10.1107/S1600536812001651/wm2582Isup2.hkl

Additional supplementary materials:  crystallographic information; 3D view; checkCIF report

## Figures and Tables

**Table 1:** Selected bond lengths (Å)

Ni1—N5	2.0865 (16)
Ni1—N4	2.1099 (15)
Ni1—N1	2.1124 (16)
Ni1—N2	2.1364 (15)
Ni1—N3	2.1491 (16)
Ni1—N6	2.1634 (15)

**Table 2:** Hydrogen-bond geometry (Å, °)

*D*—H⋯*A*	*D*—H	H⋯*A*	*D*⋯*A*	*D*—H⋯*A*
N2—H1*N*2⋯O3^i^	0.92	2.47	3.324 (2)	155
N2—H2*N*2⋯O2^ii^	0.92	2.25	3.084 (2)	150
N3—H1*N*3⋯S2^iii^	0.92	2.68	3.5921 (18)	169
N4—H1*N*4⋯O2^ii^	0.92	2.09	2.971 (2)	161
N4—H2*N*4⋯O2^iii^	0.92	2.25	3.151 (2)	165
N4—H2*N*4⋯S2^iii^	0.92	3.21	3.8838 (17)	131
N5—H1*N*5⋯O1^iii^	0.92	2.40	3.244 (2)	153
N5—H1*N*5⋯O2^iii^	0.92	2.48	3.289 (2)	147
N5—H2*N*5⋯O4^ii^	0.92	2.09	2.997 (3)	168
N6—H1*N*6⋯O3^i^	0.92	2.30	3.062 (2)	140
N6—H2*N*6⋯O1	0.92	2.44	3.307 (2)	158
N6—H2*N*6⋯S2	0.92	2.89	3.6214 (17)	137
O4—H2*O*4⋯S2	0.84	2.56	3.390 (2)	171
O5—H1*O*5⋯O3	0.84	2.02	2.858 (3)	179

Symmetry codes: (i) 
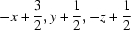
; (ii) 
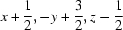
; (iii) 
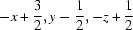
.

**Fig. 1.**
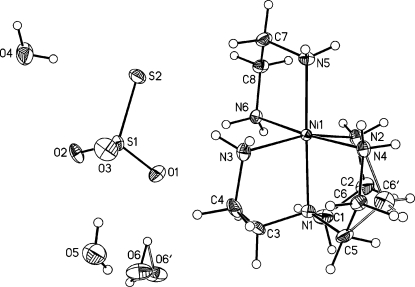
The molecular parts of the crystal structure of the title compound with atom labelling and displacement ellipsoids drawn at the 50% probability level. The disorder of the O6 and C6 atoms is shown with bonds as open lines.

**Fig. 2.**
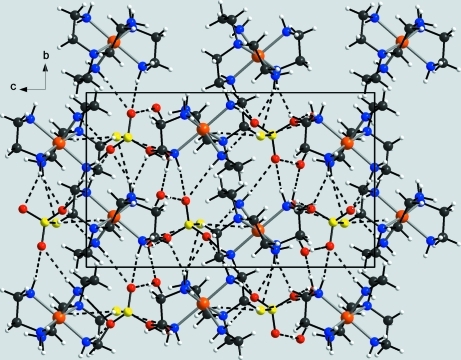
Crystal structure of the title compound with view along the b axis. Hydrogen bonding is shown as dashed lines; disordered atoms are not shown for clarity. Colour code: Ni: orange; N: blue; O: red; C: black; H white.

## supplementary crystallographic information

### Comment

The structure of the title compound was determined within a project on the synthesis, structure determination and investigation of the properties of new thioantimonates (Seidlhofer *et al.*, 2011; Lühmann *et al.*, 2011). The crystals were obtained accidentally by the reaction of [Ni(en)_3_]Cl_2_ with Sb and S in tris(2-aminoethyl)amine.

The structure consists of a discrete [Ni(tren)(en)]^2+^ complex, a thiosulfate anion and three water molecules. The Ni^2+^ cation is octahedrally coordinated by four N atoms from one tris(2-aminoethyl)amine and two N atoms from one ethylenediamine ligand (Fig. 1). The Ni—N bond lengths are between 2.0865 (16) and 2.1634 (15) Å and the N—Ni—N angles range from 82.39 (6) to 175.52 (6) °. In the crystal structure the cations, anions and water molecules are connected by a complex hydrogen-bonded network into a three-dimensional structure (Tab. 1 and Fig. 2). It is noted that only a few isolated thiosulfate nickel complexes are known up to date (Nardelli & Coghi, 1958; Varand *et al.*, 1967; Freire *et al.*, 2000; Díaz de Vivar *et al.*, 2007).

### Experimental

The title compound was synthesized by heating [Ni(en)_3_]Cl_2_ (1.4 mmol), Sb (1.4 mmol) and S (4 mmol) in 50% tris(2-aminoethyl)amine (6.6 ml) for 8 d at 413 K. The reaction mixture was cooled down, filtered off and washed with water, ethanol and acetone. The pink crystals are unstable in air. Yield: 17% based on Ni. Elemental analysis found: C 23.75%, H 7.76%, N 19.90%; calc.: C 22.28%, H 7.48%, N 19.49%.

### Refinement

The C—H and N—H hydrogen atoms were positioned with idealized geometry and were refined with U_iso_(H) = 1.2U_eq_(C, N). The O—H hydrogen atoms were located in difference maps, and their bonds lengths were set to ideal values and subsequently refined with (U_iso_(H) = 1.5 U_eq_(O) using a riding model. The C atom C6 and the O atom O6 are disordered and were refined using a split model. The C atom of lower occupancy (occupancy ratio 0.85:0.15) was refined only isotropically, whereas both O atoms (occupancy ratio 0.60:0.40) were refined anisotropically.

### Crystal data

**Table:**

[Ni(C_2_H_8_N_2_)(C_6_H_18_N_4_)]S_2_O_3_·3H_2_O	*F*(000) = 920
*M**_r_* = 431.23	*D*_x_ = 1.579 Mg m^−^^3^
Monoclinic, *P*2_1_/*n*	Mo *K*α radiation, λ = 0.71073 Å
Hall symbol: -P 2yn	Cell parameters from 4358 reflections
*a* = 10.890 (2) Å	θ = 2.4–28.1°
*b* = 10.0494 (17) Å	µ = 1.34 mm^−^^1^
*c* = 16.689 (3) Å	*T* = 170 K
β = 96.68 (2)°	Parallelepiped, pink
*V* = 1813.9 (6) Å^3^	0.16 × 0.11 × 0.06 mm
*Z* = 4	

### Data collection

**Table:**

Stoe IPDS-1 diffractometer	4358 independent reflections
Radiation source: fine-focus sealed tube	3924 reflections with *I* > 2σ(*I*)
graphite	*R*_int_ = 0.065
φ scans	θ_max_ = 28.1°, θ_min_ = 2.4°
Absorption correction: numerical (*X-SHAPE*; Stoe & Cie, 1998)	*h* = −14→14
*T*_min_ = 0.559, *T*_max_ = 0.748	*k* = −13→13
23884 measured reflections	*l* = −22→22

### Refinement

**Table:**

Refinement on *F*^2^	Secondary atom site location: difference Fourier map
Least-squares matrix: full	Hydrogen site location: inferred from neighbouring sites
*R*[*F*^2^ > 2σ(*F*^2^)] = 0.037	H-atom parameters constrained
*wR*(*F*^2^) = 0.099	*w* = 1/[σ^2^(*F*_o_^2^) + (0.0672*P*)^2^ + 0.6394*P*] where *P* = (*F*_o_^2^ + 2*F*_c_^2^)/3
*S* = 1.05	(Δ/σ)_max_ = 0.001
4358 reflections	Δρ_max_ = 0.55 e Å^−^^3^
216 parameters	Δρ_min_ = −0.77 e Å^−^^3^
2 restraints	Extinction correction: *SHELXL97* (Sheldrick, 2008), Fc^*^=kFc[1+0.001xFc^2^λ^3^/sin(2θ)]^-1/4^
Primary atom site location: structure-invariant direct methods	Extinction coefficient: 0.0202 (16)

### Special details

**Table:**

Geometry. All e.s.d.'s (except the e.s.d. in the dihedral angle between two l.s. planes) are estimated using the full covariance matrix. The cell e.s.d.'s are taken into account individually in the estimation of e.s.d.'s in distances, angles and torsion angles; correlations between e.s.d.'s in cell parameters are only used when they are defined by crystal symmetry. An approximate (isotropic) treatment of cell e.s.d.'s is used for estimating e.s.d.'s involving l.s. planes.
Refinement. Refinement of *F*^2^ against ALL reflections. The weighted *R*-factor *wR* and goodness of fit *S* are based on *F*^2^, conventional *R*-factors *R* are based on *F*, with *F* set to zero for negative *F*^2^. The threshold expression of *F*^2^ > σ(*F*^2^) is used only for calculating *R*-factors(gt) *etc*. and is not relevant to the choice of reflections for refinement. *R*-factors based on *F*^2^ are statistically about twice as large as those based on *F*, and *R*- factors based on ALL data will be even larger.

### Fractional atomic coordinates and isotropic or equivalent isotropic displacement parameters (Å^2^)

**Table:**

	*x*	*y*	*z*	*U*_iso_*/*U*_eq_	Occ. (<1)
Ni1	0.825612 (19)	0.70992 (2)	0.095341 (12)	0.00955 (10)	
N1	0.66319 (15)	0.77843 (15)	0.02610 (9)	0.0145 (3)	
C1	0.68958 (19)	0.91729 (19)	0.00384 (13)	0.0231 (4)	
H1A	0.6888	0.9758	0.0515	0.028*	
H1B	0.6249	0.9488	−0.0385	0.028*	
C2	0.8153 (2)	0.9243 (2)	−0.02702 (13)	0.0260 (4)	
H2A	0.8116	0.8783	−0.0797	0.031*	
H2B	0.8374	1.0185	−0.0351	0.031*	
N2	0.91090 (15)	0.86132 (16)	0.03095 (10)	0.0168 (3)	
H1N2	0.9468	0.9241	0.0664	0.020*	
H2N2	0.9716	0.8247	0.0039	0.020*	
C3	0.56558 (18)	0.7694 (2)	0.08050 (12)	0.0200 (4)	
H3A	0.4838	0.7849	0.0493	0.024*	
H3B	0.5790	0.8390	0.1226	0.024*	
C4	0.56718 (18)	0.6330 (2)	0.12019 (12)	0.0216 (4)	
H4A	0.5119	0.6334	0.1633	0.026*	
H4B	0.5362	0.5654	0.0797	0.026*	
N3	0.69498 (15)	0.59804 (16)	0.15526 (9)	0.0170 (3)	
H1N3	0.7082	0.5084	0.1490	0.020*	
H2N3	0.7052	0.6171	0.2095	0.020*	
C5	0.63530 (14)	0.69377 (15)	−0.04694 (9)	0.0204 (4)	
H5A	0.6615	0.7413	−0.0941	0.024*	
H5B	0.5448	0.6800	−0.0571	0.024*	
C6	0.69987 (14)	0.55683 (15)	−0.03937 (9)	0.0174 (4)	0.85
H6A	0.6530	0.4959	−0.0076	0.021*	0.85
H6B	0.7018	0.5177	−0.0937	0.021*	0.85
C6'	0.7426 (12)	0.6239 (15)	−0.0703 (8)	0.029 (3)*	0.15
H6C	0.7902	0.6853	−0.1013	0.034*	0.15
H6D	0.7142	0.5488	−0.1061	0.034*	0.15
N4	0.82558 (15)	0.57175 (15)	0.00015 (9)	0.0151 (3)	
H1N4	0.8765	0.6013	−0.0364	0.018*	
H2N4	0.8548	0.4909	0.0200	0.018*	
N5	0.98383 (15)	0.62798 (15)	0.15913 (9)	0.0155 (3)	
H1N5	0.9769	0.5368	0.1607	0.019*	
H2N5	1.0523	0.6490	0.1341	0.019*	
C7	0.99714 (19)	0.6825 (2)	0.24186 (11)	0.0197 (4)	
H7A	1.0827	0.6690	0.2678	0.024*	
H7B	0.9399	0.6364	0.2746	0.024*	
C8	0.96731 (18)	0.8296 (2)	0.23676 (11)	0.0183 (4)	
H8A	0.9727	0.8678	0.2917	0.022*	
H8B	1.0280	0.8761	0.2069	0.022*	
N6	0.84107 (15)	0.84901 (15)	0.19495 (9)	0.0139 (3)	
H1N6	0.8306	0.9350	0.1765	0.017*	
H2N6	0.7826	0.8313	0.2291	0.017*	
S1	0.59787 (4)	0.75756 (4)	0.35625 (3)	0.01400 (12)	
S2	0.77905 (5)	0.75258 (6)	0.39543 (3)	0.02618 (14)	
O1	0.58163 (15)	0.83268 (16)	0.27970 (9)	0.0263 (3)	
O2	0.53275 (14)	0.82554 (16)	0.41669 (9)	0.0241 (3)	
O3	0.55239 (16)	0.62002 (14)	0.34450 (11)	0.0307 (4)	
O4	0.70135 (19)	0.76209 (19)	0.58623 (12)	0.0412 (5)	
H1O4	0.7540	0.7212	0.6179	0.062*	
H2O4	0.7245	0.7692	0.5401	0.062*	
O5	0.31217 (17)	0.58765 (18)	0.25772 (12)	0.0389 (4)	
H1O5	0.3829	0.5982	0.2828	0.058*	
H2O5	0.2956	0.6611	0.2350	0.058*	
O6	0.3335 (2)	0.8638 (3)	0.21164 (18)	0.0350 (9)	0.60
H1O6	0.2996	0.9412	0.2226	0.053*	
H2O6	0.4147	0.8758	0.2313	0.053*	
O6'	0.3485 (2)	0.9043 (3)	0.19116 (18)	0.0382 (16)	0.40

### Atomic displacement parameters (Å^2^)

**Table:**

	*U*^11^	*U*^22^	*U*^33^	*U*^12^	*U*^13^	*U*^23^
Ni1	0.00967 (15)	0.00919 (14)	0.00982 (14)	−0.00134 (7)	0.00127 (8)	−0.00001 (7)
N1	0.0134 (8)	0.0142 (7)	0.0157 (7)	0.0003 (5)	0.0006 (6)	0.0004 (5)
C1	0.0239 (10)	0.0156 (8)	0.0283 (10)	0.0034 (7)	−0.0032 (8)	0.0080 (7)
C2	0.0295 (11)	0.0240 (10)	0.0241 (10)	−0.0055 (8)	0.0023 (8)	0.0131 (8)
N2	0.0172 (8)	0.0159 (7)	0.0185 (7)	−0.0036 (6)	0.0073 (6)	0.0010 (6)
C3	0.0108 (9)	0.0257 (9)	0.0238 (9)	0.0023 (7)	0.0029 (7)	−0.0019 (7)
C4	0.0149 (9)	0.0245 (9)	0.0269 (10)	−0.0074 (7)	0.0090 (7)	−0.0023 (7)
N3	0.0198 (8)	0.0151 (7)	0.0169 (7)	−0.0048 (6)	0.0056 (6)	0.0011 (5)
C5	0.0203 (10)	0.0245 (9)	0.0146 (8)	−0.0002 (7)	−0.0051 (7)	−0.0023 (7)
C6	0.0174 (10)	0.0182 (10)	0.0157 (9)	−0.0038 (8)	−0.0015 (7)	−0.0061 (8)
N4	0.0177 (8)	0.0131 (7)	0.0152 (7)	−0.0003 (6)	0.0041 (5)	−0.0034 (5)
N5	0.0153 (7)	0.0126 (7)	0.0180 (7)	0.0011 (6)	−0.0007 (6)	−0.0018 (5)
C7	0.0206 (10)	0.0228 (9)	0.0144 (8)	0.0027 (8)	−0.0033 (7)	−0.0017 (7)
C8	0.0179 (9)	0.0190 (8)	0.0167 (8)	−0.0012 (7)	−0.0038 (7)	−0.0056 (7)
N6	0.0170 (7)	0.0118 (6)	0.0130 (7)	−0.0006 (5)	0.0023 (5)	−0.0023 (5)
S1	0.0159 (2)	0.0109 (2)	0.0162 (2)	0.00063 (15)	0.00616 (16)	−0.00066 (15)
S2	0.0154 (3)	0.0291 (3)	0.0348 (3)	0.0045 (2)	0.0061 (2)	0.0132 (2)
O1	0.0286 (8)	0.0301 (8)	0.0198 (7)	0.0012 (6)	0.0007 (6)	0.0067 (6)
O2	0.0212 (7)	0.0251 (7)	0.0287 (7)	0.0008 (6)	0.0141 (6)	−0.0079 (6)
O3	0.0346 (9)	0.0130 (6)	0.0446 (10)	−0.0050 (6)	0.0046 (7)	−0.0052 (6)
O4	0.0467 (12)	0.0387 (9)	0.0426 (10)	0.0195 (9)	0.0234 (9)	0.0145 (8)
O5	0.0305 (9)	0.0374 (9)	0.0483 (10)	0.0004 (7)	0.0025 (8)	0.0186 (8)
O6	0.0327 (18)	0.0252 (14)	0.0431 (18)	−0.0002 (12)	−0.0129 (14)	−0.0037 (14)
O6'	0.024 (2)	0.050 (3)	0.038 (3)	0.008 (2)	−0.0087 (19)	−0.028 (3)

### Geometric parameters (Å, °)

**Table:**

Ni1—N5	2.0865 (16)	C6—H6A	0.9900
Ni1—N4	2.1099 (15)	C6—H6B	0.9900
Ni1—N1	2.1124 (16)	C6'—N4	1.492 (12)
Ni1—N2	2.1364 (15)	C6'—H6C	0.9900
Ni1—N3	2.1491 (16)	C6'—H6D	0.9900
Ni1—N6	2.1634 (15)	N4—H1N4	0.9200
N1—C3	1.479 (2)	N4—H2N4	0.9200
N1—C1	1.481 (2)	N5—C7	1.477 (2)
N1—C5	1.489 (2)	N5—H1N5	0.9200
C1—C2	1.519 (3)	N5—H2N5	0.9200
C1—H1A	0.9900	C7—C8	1.514 (3)
C1—H1B	0.9900	C7—H7A	0.9900
C2—N2	1.479 (3)	C7—H7B	0.9900
C2—H2A	0.9900	C8—N6	1.480 (2)
C2—H2B	0.9900	C8—H8A	0.9900
N2—H1N2	0.9200	C8—H8B	0.9900
N2—H2N2	0.9200	N6—H1N6	0.9200
C3—C4	1.521 (3)	N6—H2N6	0.9200
C3—H3A	0.9900	S1—O2	1.4675 (14)
C3—H3B	0.9900	S1—O3	1.4736 (15)
C4—N3	1.488 (3)	S1—O1	1.4767 (15)
C4—H4A	0.9900	S1—S2	2.0058 (8)
C4—H4B	0.9900	O4—H1O4	0.8401
N3—H1N3	0.9200	O4—H2O4	0.8400
N3—H2N3	0.9200	O5—H1O5	0.8400
C5—C6'	1.454 (12)	O5—H2O5	0.8401
C5—C6	1.5439	O6—H1O6	0.8898
C5—H5A	0.9900	O6—H2O6	0.9142
C5—H5B	0.9900	O6'—H1O6	0.8737
C6—N4	1.457 (2)	O6'—H2O6	0.9683
			
N5—Ni1—N4	92.99 (6)	C6—C5—H5A	108.9
N5—Ni1—N1	175.52 (6)	C6'—C5—H5B	134.5
N4—Ni1—N1	82.86 (6)	N1—C5—H5B	108.9
N5—Ni1—N2	98.99 (6)	C6—C5—H5B	108.9
N4—Ni1—N2	92.98 (6)	H5A—C5—H5B	107.7
N1—Ni1—N2	83.00 (6)	N4—C6—C5	109.90 (8)
N5—Ni1—N3	96.21 (6)	N4—C6—H6A	109.7
N4—Ni1—N3	93.66 (6)	C5—C6—H6A	109.7
N1—Ni1—N3	82.39 (6)	N4—C6—H6B	109.7
N2—Ni1—N3	163.06 (6)	C5—C6—H6B	109.7
N5—Ni1—N6	82.94 (6)	H6A—C6—H6B	108.2
N4—Ni1—N6	175.51 (6)	C5—C6'—N4	113.0 (9)
N1—Ni1—N6	101.25 (6)	C5—C6'—H6C	109.0
N2—Ni1—N6	85.81 (6)	N4—C6'—H6C	109.0
N3—Ni1—N6	88.69 (6)	C5—C6'—H6D	109.0
C3—N1—C1	112.73 (16)	N4—C6'—H6D	109.0
C3—N1—C5	112.01 (14)	H6C—C6'—H6D	107.8
C1—N1—C5	111.11 (14)	C6—N4—Ni1	109.06 (10)
C3—N1—Ni1	105.07 (11)	C6'—N4—Ni1	108.0 (5)
C1—N1—Ni1	105.65 (11)	C6—N4—H1N4	109.9
C5—N1—Ni1	109.87 (11)	Ni1—N4—H1N4	109.9
N1—C1—C2	109.73 (16)	C6—N4—H2N4	109.9
N1—C1—H1A	109.7	C6'—N4—H2N4	138.3
C2—C1—H1A	109.7	Ni1—N4—H2N4	109.9
N1—C1—H1B	109.7	H1N4—N4—H2N4	108.3
C2—C1—H1B	109.7	C7—N5—Ni1	108.42 (12)
H1A—C1—H1B	108.2	C7—N5—H1N5	110.0
N2—C2—C1	110.62 (16)	Ni1—N5—H1N5	110.0
N2—C2—H2A	109.5	C7—N5—H2N5	110.0
C1—C2—H2A	109.5	Ni1—N5—H2N5	110.0
N2—C2—H2B	109.5	H1N5—N5—H2N5	108.4
C1—C2—H2B	109.5	N5—C7—C8	108.22 (15)
H2A—C2—H2B	108.1	N5—C7—H7A	110.1
C2—N2—Ni1	108.64 (12)	C8—C7—H7A	110.1
C2—N2—H1N2	110.0	N5—C7—H7B	110.1
Ni1—N2—H1N2	110.0	C8—C7—H7B	110.1
C2—N2—H2N2	110.0	H7A—C7—H7B	108.4
Ni1—N2—H2N2	110.0	N6—C8—C7	109.67 (15)
H1N2—N2—H2N2	108.3	N6—C8—H8A	109.7
N1—C3—C4	110.49 (16)	C7—C8—H8A	109.7
N1—C3—H3A	109.6	N6—C8—H8B	109.7
C4—C3—H3A	109.6	C7—C8—H8B	109.7
N1—C3—H3B	109.6	H8A—C8—H8B	108.2
C4—C3—H3B	109.6	C8—N6—Ni1	105.19 (11)
H3A—C3—H3B	108.1	C8—N6—H1N6	110.7
N3—C4—C3	110.30 (15)	Ni1—N6—H1N6	110.7
N3—C4—H4A	109.6	C8—N6—H2N6	110.7
C3—C4—H4A	109.6	Ni1—N6—H2N6	110.7
N3—C4—H4B	109.6	H1N6—N6—H2N6	108.8
C3—C4—H4B	109.6	O2—S1—O3	110.26 (10)
H4A—C4—H4B	108.1	O2—S1—O1	109.74 (10)
C4—N3—Ni1	109.44 (11)	O3—S1—O1	111.09 (10)
C4—N3—H1N3	109.8	O2—S1—S2	108.85 (7)
Ni1—N3—H1N3	109.8	O3—S1—S2	108.82 (8)
C4—N3—H2N3	109.8	O1—S1—S2	108.01 (7)
Ni1—N3—H2N3	109.8	H1O4—O4—H2O4	111.2
H1N3—N3—H2N3	108.2	H1O5—O5—H2O5	104.5
C6'—C5—N1	113.6 (6)	H1O6—O6—H2O6	102.6
N1—C5—C6	113.25 (8)	H1O6—O6'—H2O6	99.6
N1—C5—H5A	108.9		

### Hydrogen-bond geometry (Å, °)

**Table:**

*D*—H···*A*	*D*—H	H···*A*	*D*···*A*	*D*—H···*A*
N2—H1N2···O3^i^	0.92	2.47	3.324 (2)	155.
N2—H2N2···O2^ii^	0.92	2.25	3.084 (2)	150.
N3—H1N3···S2^iii^	0.92	2.68	3.5921 (18)	169.
N4—H1N4···O2^ii^	0.92	2.09	2.971 (2)	161.
N4—H2N4···O2^iii^	0.92	2.25	3.151 (2)	165.
N4—H2N4···S2^iii^	0.92	3.21	3.8838 (17)	131.
N5—H1N5···O1^iii^	0.92	2.40	3.244 (2)	153.
N5—H1N5···O2^iii^	0.92	2.48	3.289 (2)	147.
N5—H2N5···O4^ii^	0.92	2.09	2.997 (3)	168.
N6—H1N6···O3^i^	0.92	2.30	3.062 (2)	140.
N6—H2N6···O1	0.92	2.44	3.307 (2)	158.
N6—H2N6···S2	0.92	2.89	3.6214 (17)	137.
O4—H2O4···S2	0.84	2.56	3.390 (2)	171.
O5—H1O5···O3	0.84	2.02	2.858 (3)	179.

Symmetry codes: (i) −*x*+3/2, *y*+1/2, −*z*+1/2; (ii) *x*+1/2, −*y*+3/2, *z*−1/2; (iii) −*x*+3/2, *y*−1/2, −*z*+1/2.
